# ‘Whose life are They Going to Save? It’s Probably Not Going to be Mine!’ Living With a Life-Shortening Condition During the Coronavirus (COVID-19) Pandemic: A Grounded Theory Study of Embodied Precarity

**DOI:** 10.1177/10497323221131692

**Published:** 2022-12

**Authors:** Sarah Earle, Maddie Blackburn, Lizzie Chambers, Julia Downing, Kate Flemming, Jamie Hale, Hannah R. Marston, Lindsay O’Dell, Valerie Sinason, Lucy Watts, Sally Whitney

**Affiliations:** 1 172243 The Open University, UK; 2 276759 Together for Short Lives, UK; 3 International Children’s Palliative Care Network, UK/South Africa; 4 University of York, UK; 5 Expert by Experience, UK; 6 Independent researcher, UK

**Keywords:** life-threatening, life-limited, end-of-life, precarity, vulnerability, young adults, disability, embodiment, health experience, illness, COVID-19

## Abstract

This article explores the experiences of young adults with a life-shortening condition in the first wave of the Coronavirus (COVID-19) pandemic in the UK. It presents the findings from an inclusive qualitative research study using constructivist grounded theory which aimed to examine the unintended consequences of pandemic control measures (lockdown and ‘shielding’) on this population. Purposive and theoretical sampling methods were used to recruit young adults with a life-shortening condition, employing a range of recruitment methods such as social media, advertising in newsletters and snowballing. Twenty-six young adults (aged 22–40 years), with a wide range of life-shortening conditions participated in the study. Seventeen participants were female and nine male. The majority identified as White British/Other and the remainder as Black British (2), Mixed Race (2) or Latin American (1). Data were generated iteratively using in-depth guided interviews and analysed collectively by an inclusive research team using the constant comparative method. The article explores a theory of embodied precariousness of living with a life-shortening condition during the first wave of the Coronavirus pandemic in relation to three categories: the rationing of life-saving treatment, the deterioration of health and retraction of healthcare provision, and the disruption of typical care arrangements. The findings show that the pandemic control measures introduced to keep people safe have intensified the precarity of this group promoting inequalities in healthcare and health outcomes. The article identifies some implications for practice to support the future management of unexpected and unwanted change.

## Introduction

This article discusses the experiences of young adults with life-shortening conditions during the first wave of the Coronavirus pandemic in 2020 in the UK. We argue that living with such a condition is a precarious embodied state and, in particular, draw on Butler’s (2004b) concept of ‘corporeal vulnerability’; a fundamental form of precariousness applicable to all human life. However, like Butler, we acknowledge that human physical vulnerability is not equally distributed creating ‘exclusionary conceptions of who is normatively human’ and ‘what counts as a liveable life’ (Butler, 2004b, p. xiv-xv). Precarity is therefore distinct from precariousness in that it refers to a politically induced state which renders some lives as more liveable than others (Butler, 2004a). As one disability scholar has argued: ‘Media stories of nonsentient or suffering people helplessly tethered to tubes and machines are the horror tales of our age’ (Gill, 2006, p. 186). During the Coronavirus pandemic this perception of disability has been laid bare and amplified.

In the UK (and in other countries) people with ‘pre-existing’ or ‘underlying’ health conditions have been casually and repeatedly referred to as those most at risk of dying from COVID-19 (Hastie, 2020; Liddiard, 2020). This discourse initially served to reassure the (healthier) population that they were not at risk but as Abrams and Abbott (2020) have pointed out, this language of pre-existing conditions serves to position disabled or vulnerable people as having a life not worth living. This othering (Ktenidis, 2020) of the most vulnerable in society has rendered the precarity of those living with life-shortening conditions simultaneously visible and invisible. They are foregrounded as part of a popular ontologically violent (Liddiard, 2020) risk discourse yet then disappear from view through othering that discounts their life.

## Life-Shortening Conditions and Corporeal Vulnerability

Living with a life-shortening condition is a unique form of precariousness as people with such conditions are particularly corporeally vulnerable. Life-shortening conditions include a wide range of heterogeneous and complex conditions including some that are extremely rare (Hain & Devins, 2011). They are broadly categorised into two main groups – life-limiting conditions where there is no reasonable hope of cure (e.g. Duchenne muscular dystrophy) and life-threatening conditions where curative treatment may exist but may fail (e.g. cancer) (TfSL, 2018). Due to advances in clinical treatments and medical care, there are now more young adults living with these conditions in the UK, and elsewhere, and this population will continue to grow (Fraser et al., 2021). Definitions of what counts as life-shortening changes over time as medical and technological advances impact on survival rates. The health and social care needs of this population are, however, increasing in complexity since the technological advances that improve survival can have significant consequences for the lives of survivors (Rempel & Harrison, 2007). As such, many young adults with life-shortening conditions have cognitive impairments and/or rely on advanced medical technologies such as ventilatory support or tube-feeding.

The vulnerability of living with a life-shortening condition takes many corporeal forms and includes being told – often from birth or a young age – that you will die at a younger age than your peers and that you will probably not live long enough to become an adult (Blackburn, 2019). It can also mean living with diagnostic uncertainty – sometimes having a condition that is rare or so complex that it has not yet been named (TfSL, 2013). People with life-shortening conditions often experience frequent bouts of both predictable and unpredictable ill-health which sometimes require long periods of hospitalisation (TfSL, 2013). Many are living with chronic pain or other disabling impairments and experience debilitating side-effects from medication or treatment regimens such as fatigue or cognitive impairment (Noyes et al., 2014). Living with this embodied precariousness often also means the curtailment of education and occupational opportunities and the subsequent economic, social and psychological consequences of this loss (TfSL, 2015; Bomba, Herrmann-Garitz & Schmidt et al., 2017). In turn this implies a reliance on (often retracting) welfare provision. This type of corporeal vulnerability then forms part of a ‘precarity trap’ (Standing, 2011) underpinned by educational, economic and social exclusion.

## Precarity, Health and Disability

The concept of precarity has been employed principally in the field of work and employment. In this context precarity is understood to be the consequence of insecure and deregulated labour markets which impact on the ability to have a predictable and coherent future (Standing, 2011). The concept of precarity is often also used to explore how deregulation of the labour market has gone hand-in-hand with a retraction of welfare within neoliberal states (Nettleton & Burrows, 2001). The protective function of the welfare state, through formal provision of social welfare, is seen as a safety net securing against some of the worse impacts of precarious labour. Precariousness is, therefore, complex and multi-faceted and based on multiple and sweeping social change.

More recently the concept of precarity has been applied to issues of health and healthcare showing how precariousness impacts on physical and mental health and how this then impacts on work and employment, so creating a cycle of precarity, or precarity trap (Standing, 2011). There is wide-ranging work within this multi-disciplinary field, and it includes analyses of precarious employment on health (e.g. McKee et al., 2017; Gray et al., 2021), in the context of austerity (Barlow, McKee & Stuckler, 2015) and COVID-19 (McNamara et al., 2021). There is also specific focus on precariousness, precarity and mental health (e.g. Moscone et al., 2016; Utzet et al., 2020; Brenman, 2021).

Other scholars have explored precarity in the sense intended by Butler, focussing on the corporeality of physical vulnerability and how this intersects with socio-economic and political drivers. For example, Mills (2017) examines the biopolitical landscape of HIV treatment in South Africa, developing the concept of embodied precarity to explore access to antiretroviral therapies within a particular socio-political context. Greiner, Lloyd and Phillipson (2017) have focussed on precarity, dementia and frailty in the UK exploring the fragility and limitations of the ageing body. Similarly, disability scholars have also explored precarity in relation to the politicisation of vulnerability (Knight, 2014) and disabled embodiment and vulnerability within neoliberalism (Shildrick, 2019). However, not all disability scholars agree with Butler’s notion of fundamental and universal vulnerability. Writers such as Gill (2006), for example, argue that: ‘disabled people are not innately vulnerable but are made so by a social environment that devalues human difference’ (p. 183). Here, we take the position that all human beings are innately vulnerable but that precarity is unequally distributed.

## Method

### Study Aim and Design

This article draws on an inclusive qualitative research study informed by constructivist grounded theory methodology (cGT) (Charmaz, 2006). There are many variations of grounded theory but cGT is based on pragmatist underpinnings (Bryant, 2009) that foreground interpretive and abstract understandings of the empirical world (Charmaz, 2017). Inclusive research is a term used that can encompass a range of approaches, but it generally refers to research that shifts the power dynamics between those who typically do research and those who are the subjects of that research (Nind, 2014; Liddiard et al., 2018). Central to both cGT and inclusive research is the principle of co-construction (Teram et al., 2005). In cGT it is assumed that data are generated through a reflexive process of co-creation between researcher and research participant (Charmaz, 2006; 2017). In inclusive research approaches, emphasis is placed on co-creation processes including the co-creation of knowledge and action (Nind, 2014; Liddiard et al., 2018). This inclusive study included three ‘experts by experience’, or co-researchers; young adults (in their 20s and 30s) with a life-shortening condition who were involved in all stages of the research.

The study aimed to investigate the unintended consequences of pandemic control measures on the experiences of young adults with life-shortening conditions during the first wave of the Coronavirus (COVID-19) pandemic in the UK. It is part of a larger study which also included interviews with family members of young adults with life-shortening conditions, whose data are not reported here.

### Research Ethics

A favourable ethical opinion was secured from The Open University Human Research Ethics Committee (No. 3595, June 2020). Participants completed an online consent form prior to taking part in the research and then consent was taken again verbally at the beginning of the interview process. During interviews, and particularly in situations where participants became distressed or seemed fatigued, the interviewer would check that the participant was happy to continue. Every participant was followed up by email within 24 hours post-interview, signposting participants to additional information and support services. Interviewers were also offered a debrief with the principal investigator (PI) post-interview. A trusted colleague with clinical experience in this field, who was not part of the research team but had worked with us on similar projects, was also on standby should further support be required for participants or interviewers and this formed part of our research protocol. Participants were offered an honorarium (£40 voucher) to thank them for their participation.

### Sampling and recruitment strategies

We initially used purposive sampling to recruit participants using agreed inclusion/exclusion criteria. Participants were considered for inclusion if they were aged between 18 and 40 years, had a life-shortening condition and were living in the UK. Using cGT methodology we went back and forth (Lincoln & Guba, 1985) between collecting and analysing data and this informed subsequent theoretical sampling of participants, where possible. However, as other researchers (Timonen et al., 2018) have acknowledged, although theoretical sampling is the gold standard within grounded theory, we encountered some constraints, notably, our sensitivity to the potential vulnerability of respondents and the practicalities of working in tandem and within a large team.

We recruited for six months (June–December 2020). Recruitment methods were varied and included using social media as well as advertising in the online bulletins and newsletters of the project’s partner organisations (*Hospice UK*, *International Children's Palliative Care Network* and *Together for Short Lives*). Project team members used their wider networks to reach out to organisations and professional colleagues working in relevant sectors. Our co-researchers used snowballing to reach out to other young people they knew with life-shortening conditions.

### Participants

We recruited 26 people with life-shortening conditions, aged 22–40 years. Seventeen participants were female and nine male. Eighteen participants identified as White British or White Other, three as British Asian, two as Black British, two as mixed race (White Asian) and one as Latin American. Young people reported a wide range of conditions including neurodegenerative conditions, fatal respiratory disorders, multiple organ failure, rare genetic conditions of the connective tissues or of the bones, autoimmune disorders, cancers and diseases so rare and complex that they were (as yet) undiagnosed but known to be progressive and assumed to be fatal. Given that many of these conditions are very rare we have sought to protect the identity of research participants by not identifying specific disorders.

### Data Generation

Data were generated via in-depth interviews which took place between July and December 2020 using a topic guide. A commitment to going back and forth between data collection and analysis meant that we continued to reframe the guide as the study progressed so that we could refine and check our emergent analysis and nascent theory (Charmaz, 2014). Most participants were interviewed independently but four chose to be interviewed together either with a parent (who also participated in the study, but whose data are not the focus here) or sibling. Two participants who could not communicate orally asked to take part and we accommodated this by adapting the topic guide using an email-interview method (Benford & Standen, 2011). The project team agreed that this yielded less-rich data (in comparison to other interviews) but that our commitment to inclusive research with this population required flexibility (Topping et al., 2021). The multi-disciplinary project team consisted of four academic researchers, three co-researchers and four advisors with policy and/or practice expertise; one of the academic researchers had a policy and practice background and three of the advisors also had experience of academic research. All the academic researchers and two of the co-researchers carried out interviews. Interviewers sometimes worked in pairs because some members of the team were not experienced qualitative researchers (e.g. the co-researchers) or, were experienced, but had not previously conducted research with this population group (two of the four academic researchers). All interviewers recorded their observations of the interview in a field note or memo which were used in analysis. The interviews took place using a video-conferencing platform and they were recorded with consent. Length of interviews varied from 59 minutes to two hours and 15 minutes but most interviews were well over one hour and some interviews were carried out over two sessions to accommodate the health needs of participants. The interviews were transcribed verbatim by a professional transcription service with a comprehensive security management policy using secure file transfer servers and SSL encryption. Culturally appropriate pseudonyms were assigned prior to transcription to ensure participant anonymity and then transcribed files were anonymised further by replacing identifiable text with meaningful descriptive text or tags.

### Data Analysis and Theorising

Data were analysed using the constant comparative method (Glaser & Strauss, 1967). The project team met regularly to discuss interview transcripts and continued to meet until all the transcripts had been analysed. Before each coding meeting, every team member would read, re-read and code each transcript and share their coding. Members were asked to line-by-line code using gerunds; doing words that help illicit ‘what is going on?’ in the data (Charmaz, 2014) and to memo around that analysis. Examples of initial codes include: ‘not wanting to die’ and ‘feeling invisible’. These meetings were recorded and auto-generated transcripts used as a form of memoing, alongside additional brief meeting notes. Following this, members would send their coding and memos to the PI who would use these to refine codes going forward, developing more focused and then theoretical coding based on this initial coding stage (Charmaz, 2014) as the study progressed. Examples of focused codes include: ‘pivotal moments of realisation’ and ‘negotiating care’. Data analysis was supported by using NVivo 12 and all transcripts, analysis documents and memos were incorporated into NVivo so that they could be searched and organised as part of the process of analysis. The PI would regularly share the NVivo code book with the team during the process of analysis but members were encouraged to freely engage with the data according to their own interpretive understandings of participants’ subjective experiences in acknowledgement that: ‘Codes do not reflect inherent truths. Instead they reflect what we see and define at a particular point in time…’ (Charmaz, 2017, p. 3). Through a process of interpretation that recognises the role of the researcher in generating, analysing and theorising data, we then set out to make sense of the participants’ experiences of pandemic control measures and their unintended consequences by developing analytical categories. The categories discussed below were generated inductively through a close reading of the data that seeks to represent the views of participants as integral to the process of analysis and theory-building (Charmaz, 2014).

## Embodied Precarity in Experiences of Shielding and Lockdown

A theory of embodied precarity refers to the constant existential and material threat experienced by young adults with life-shortening conditions as they lived through the consequences of pandemic control measures. This threat was experienced in relation to the following categories: (1) the rationing of life-saving treatment, (2) the deterioration of health and retraction of healthcare provision and (3) the disruption of typical care arrangements. Embodied precarity describes the fundamental corporeal vulnerability inherent in living with a life-shortening condition and its relationship to wider socio-political conditions and interdependencies.

### Rationing of Life-Saving Treatment

Many participants were distressed about the possibility that life-saving treatment would be withheld should they become ill with COVID. Megan, who has a complex undiagnosed disorder, noted: ‘*Whose life are they going to save? It’s probably not going to be mine.*’ In the UK, guidance was issued on protecting people defined on medical grounds as ‘extremely clinically vulnerable’ (Abrams & Abbott, 2020). Those defined as such were sent a letter advising them to ‘shield’ (stay at home and minimise face-to-face interactions). Most participants did not receive their letter promptly and often sought advice from their doctor. Exchanges with health providers made it clear they would not be prioritised in a health emergency. Bashir, who has a muscle-wasting condition was told that, ‘*if you do get ill we’d recommend you don’t go to hospital, and get in touch with us and we’ll try and help you the best we can*.’

For some individuals, public debates about the rationing of healthcare and who would be prioritised for treatment during the pandemic heightened their sense of embodied vulnerability. Laura, who has a neurological condition, said:

‘… there was quite a lot of buzz about if there weren’t enough ventilators to go around that disabled people wouldn’t get them. … I would be seen as the one who didn’t deserve it. I’d never really thought about that before..… it’s kind of worrying that people with long term health conditions have kind of been almost like shoved to one side.’

Indeed, many of the participants spoke about being ‘*forgotten’* or ‘*abandoned’* during the pandemic. Laura had never seen herself as ‘vulnerable’ and describes her feelings of terror:

‘Only since this and I’ve realised … how weak I actually am. I normally just get on with my life … I don’t think I’m any different to anyone else in a way. Obviously now I realise I am. You know, the thought of getting coronavirus… they wouldn’t give me a ventilator, they’d give it to the stronger person. I literally cried about that for days … that was terrifying, absolutely terrifying.'

An understanding of their corporeal vulnerability, coupled with the precarity of needing emergency treatment during the pandemic provided many participants with strong motivation to shield and avoid the virus. Debbie, who has a neuromuscular disorder said:

‘… as it went on I got more and more afraid. Because I heard on Twitter and things about other people who are disabled having DNRs attached to their names and basically people dying or not getting treatment. And I was like right, well I really cannot get this…. I don’t want to die basically.’

Eventually most participants in the study received at least one letter which identified them as extremely clinically vulnerable. Receiving a letter that spelt out their vulnerability and asked them to shield for their own protection brought into sharper reality an already precarious existence. Bethany, who has cancer, expressed this clearly:

‘… your health is very precarious, but to have these things actually on paper really makes it very concrete…. Especially then when that category is talked about in the news that people only die if they have underlying health conditions, it doesn’t matter and it’s only extremely vulnerable people.’

She refers to the underlying narrative that othered vulnerable people (Ktenidis, 2020). The data from this study show how early in the pandemic there was a moment of self-identification with this othering process. Jasmine, who has multiple organ failure, said:

*‘*Myimmediatereaction,it’snotalright*. *I’moneof**them** [emphasis in original].*’*

In light of this self-identification, participants often spoke about whether they were ‘*viable’*, ‘*worthy’*, ‘*important’* or ‘*deserving’* enough to save in comparison to others. Umendra, who has a life-threatening bone disorder said:

‘… early on in the crisis when there were ventilator shortages and stuff and people had to make decisions who got them and who didn’t. I knew that … I wouldn’t be prioritised. And that really hit me quite hard.’

The possibility that life-saving treatment would be withheld during the pandemic posed both a material and existential threat to the embodied precariousness of the participants in the study.

### Deterioration of health and retracting healthcare provision

Many participants believed that they had experienced a deterioration in their physical and/or mental health because of the pandemic. For some participants regular exercise (such as walking) was a crucial component of staying healthy and the need to shield severely restricted them. Some participants acknowledged that due to the degenerative nature of their condition, their physical health would have deteriorated anyway but felt that the consequences of lockdown and shielding had affected them nonetheless. Debbie said:

‘Yeah, so my health was already deteriorating anyway, and I’ve not done as much exercise and I’m not moving as much, because I’m not physically out and about, so I know that has probably affected it.’

Participants also talked about how the pandemic had seriously impacted on their mental wellbeing. They often talked about ‘*overthinking’* or having ‘*too much time*’ and that this impacted negatively on them. Cissy, who has a complex connective tissue disorder, spoke about why she had been feeling so down:

‘I had quite an active social life prior to having to shield.... I was in two choirs. I went to a board game club. I did some art therapy, stuff like that…. And I was quite involved with the church. And to have that social contact just stopped abruptly I found that quite difficult to just not see people....’

In some cases, individuals had pre-existing mental health issues which they were typically able to manage effectively but lockdown and shielding had exacerbated symptoms. Ajay, who is autistic and has obsessive behaviours and compulsive thoughts, in addition to a rare neurological disorder, spoke about how the pandemic had worsened his symptoms:

‘My hands are all broken and cracked because I’ve been washing them so much at the start of lockdown when I was in the house. I was so scared, mum had to tell me to stop because my hands were burned…. I thought yeah, I just kept asking if I was going to die….’

Sian, who told us about his experience of depression following the death of his younger brother from the same neurodegenerative condition, explained how he had needed to ask his doctor for anti-depressants even though he had previously avoided them:

‘I always thought antidepressants were a weakness. But obviously lockdown came about. I thought right I’ve got to do something about this, and I took them….’

Many participants felt that their physical and/or mental health had deteriorated because of pandemic control measures. Most participants also spoke about some retraction of healthcare provision during the pandemic. For some this meant that routine appointments were cancelled or that face-to-face care moved to telephone appointments. These changes did not always have an immediate impact on health, but they were typically not welcomed. Kadeem, who has a neurodegenerative condition said that: ‘*It has been very stressful. Not being able to see a consultant for a face-to-face consultation is hard*…*.*’

Other participants were much more affected by the deterioration in healthcare provision. They spoke about how their healthcare needs were no longer being met because of the pandemic and how this meant that their life was ‘*on hold’*. Bethany said:

‘…everything has been pretty much suspended.… all my survivorship programme … as a transplant patient I don’t have an immune system to myself and that had only just started before COVID so that’s been on pause…’

While participants typically acknowledged that their health was likely to deteriorate over time, several believed that the absence of healthcare normally available to them was contributing to their decline. Isla, who has a neuromuscular disorder said:

‘… I found out that my lung function was much worse than I thought… It’s a progressive thing, so over time they will get worse…. But obviously if I’d known about the lung function back in March I’d have tried to get that sorted. I was locked down.’

Most young adults with life-shortening conditions have complex needs that require ongoing treatment. Those with rare and undiagnosed disorders require particularly active management to investigate symptoms. Jasmine spoke about how the active investigation of her unusual symptoms had stopped:

‘I might possibly have a condition that is rare and undiagnosed …. because of COVID a lot of the investigations and hospice care, palliative care, advanced care planning has all been put on hold….’

Participants made it clear that having healthcare on hold impacted on their day-to-day lives in very significant ways; both materially and existentially. Participants often said that they felt ‘*invisible’*; they wanted others to ‘*acknowledge’* them and wanted their death to ‘*count’*. Chrissie, who has multiple complex disorders, said:

‘We don’t stop being sick just because there is a pandemic. So when all the hospitals locked down to everybody else what are we supposed to do in the meantime? Why did our health have to be put on pause? How many people have died because they didn’t get treatment that they needed…? It’s not fair.’

The gradual deterioration of health can be characteristic of those living with a life-shortening condition combined with the unintended consequences of pandemic control measures increased the precariousness for the participants of this study. This was then compounded by a lack of access to routine healthcare for a population that typically already makes disproportionate use of services based on high needs.

### Disruption of Typical Care Arrangements

Many participants spoke about how worried they had been at the beginning of the pandemic that their typical care arrangements would be severely disrupted and that this would put their health at risk. In some instances, this fear was not realised but for many participants managing care during lockdown was highly problematic, requiring constant oversight, management and negotiation.

Cissy, who typically lives independently, describes how she was refused care during lockdown and had to draw on her mother for support. The discussion is framed around not wanting to become too dependent and on concerns for her mother’s own physical vulnerability as she ages:

‘I’m hoping once the pandemic is more settled and over that I can scale it back again, because I’m aware that she’s ageing and that I don’t want to become too dependent on her.’

Worries about the future were commonly discussed and these concerns were echoed by other participants for whom the pandemic had heightened anxieties about who would care for them should one or both parents die. Debbie lives with both parents and her brother (who has the same progressive neuromuscular disorder, but who did not participate in the study) asked ‘*I mean if they both died at the same time, what would happen?*’

For other participants, parents formed part of a regular team of carers. Megan, who lives with her mother, describes how she negotiated with care staff to manage Coronavirus infection risk, while relying more on her mother for care:

‘So my staff will text me and say, “my daughter’s come down with a bit of a cold tonight, do you want me to come in?” And I’ve said, “no can you please keep away and unless they had a negative COVID test”. So it’s been a collaborative process between me and my staff… And obviously because of my mum being able to step in, even if I don’t have care here, I’ve not been left without care…’

Participants frequently spoke about how ‘*grateful’* and ‘*thankful’* they were of the carers that continued to work with them and frequently acknowledged the ‘*sacrifices’* that those carers made to ensure their safety which included limiting some of their (other) work, not spending time with friends and family, and restricting time spent in the outside world. Participants often commended carers for keeping them safe and for allowing them to remain independent from family.

The pandemic control measures of lockdown and shielding meant staying indoors, not going out and relying on others for care, as well as for other basic needs such as food and medicines. In several instances, participants were not able to retain their independence and spoke about having to leave their own homes and temporarily relocate back to their parental home in order to meet their basic needs. Laura finds it difficult to walk and carry things and said:

‘Because I live alone, the first thing that I started to notice was that it was very difficult to get a delivery slot and that is how I rely on getting my shopping because I live on the first floor so it’s quite difficult for me to carry anything up… that’s when I started to really worry about the pandemic and eventually at the end of March I actually went to stay with my mum …’

For some, the pandemic accelerated a permanent move in location to be nearer to family. At the time of the first lockdown in England, Bethany, who lived with her partner in shared accommodation – made the decision to permanently relocate to Scotland:

‘COVID was one of the biggest pushing factors… We wanted to move back to Scotland for a while but it really just kind of fast forwarded everything....’

Young adults with life-shortening conditions were expected to shield but participants’ accounts highlight the precarious nature of living with a condition that means you are reliant on other people (and technologies) to meet basic needs vital to survival. Typical care arrangements were often disrupted during the pandemic and an increased reliance on family – not always desired – became necessary.

## Discussion

In this article, we argue that living with a life-shortening condition is a fundamentally precarious embodied state but that experiences of precarity have been amplified and magnified by the pandemic and by the measures that have been introduced to protect them and the wider population.

### Embodied Precarity

#### Rationing of Life-Saving Treatment

Concerns about the rationing of life-saving treatment provide a clear example of how the corporeal vulnerability and precarious survival (Rempel & Harrison, 2007) of young adults with life-shortening conditions are shaped by politically induced conditions (Grenier et al., 2017). Some of the participants in the study spoke readily about their condition as degenerative and progressive and about how they expected their health to deteriorate gradually as time passed, but, for others, the pandemic brought these issues into focus, sometimes for the first time. Some young adults were explicitly told by health providers that they would not receive life-saving treatment in an emergency. Others were generally aware of their metaphorical existence in public debates about the rationing of healthcare for people with pre-existing or underlying health conditions. Often, the pandemic provided a ‘moment of realisation’ (Menezes, 2010, p.41) which highlighted their vulnerability and their belonging to this category of corporeally vulnerable other (Ktenidis, 2020).

It is not known whether any of the participants in this study were ultimately denied treatment but, at the time of interviewing, the majority were concerned about this. As McKee et al. (2017, p. 4) state, ‘It is also important to note that precariousness may be perceived, even if not objectively demonstrable. An individual’s perception may be different from the reality, but is nonetheless important…’ The pandemic highlighted individual feelings of corporeal vulnerability and brought to the fore issues of precarity, inequality and unfairness. In the immediate moment, recognition of their precarious state led to shock, dismay and fear but then seemed to bring greater resolve in the face of the threat of Coronavirus as participants did what they could to avoid illness and death.

#### Deterioration of health and retraction of healthcare provision

The corporeal vulnerability of young adults with life-shortening conditions is shaped by their experiences of progressive and/or degenerative illness that cannot be cured. Most participants spoke about a decline in physical and/or mental health during the pandemic; a subject widely discussed in the media in relation to the wider population (e.g. see Daly et al., 2020) and is, therefore, unsurprising. Many of the participants in the study spoke about the eventual slow deterioration of their health, suggesting that some deterioration would have happened regardless. However, this was not the case for all participants who believed that physical deconditioning could be attributed to their experiences of shielding. The same was true for mental health. Some participants struggled with mental health prior to the pandemic – and studies suggest that there is an increased prevalence of mental health disorders such as anxiety and depression in this population (Pinquart & Shen, 2011b; 2011a; Barker, Beresford & Bland et al., 2019) – but symptoms often worsened and those who may have been medication-free prior to the pandemic were no longer medication-free subsequently.

Most young adults with life-shortening conditions rely on regular routine healthcare to manage their condition and to deal with symptoms such as chronic pain (Cook et al., 2016). All the participants spoke about cancellation of routine appointments. In some instances, this did not have an immediate impact, but most participants thought that their healthcare and health status were compromised. Life-shortening conditions are complex to manage and often require frequent periods of investigation, treatment and hospitalisation which could be vital to prolonging life (Noyes et al., 2014; Knighting et al., 2018). People with life-shortening conditions may also experience acute episodes of ill-health which require immediate treatment and could be life-threatening. Our research highlights how the reduction of routine healthcare as a result of the pandemic will have had a disproportionate effect on people living with life-shortening conditions who make routine yet habitually disproportionate use of healthcare. As Bajwah, Koffman & Hussain et al. (2021) have noted in their study of palliative care services during the pandemic, policies can have unintended adverse effects on at-risk populations creating significant inequities in health outcomes. Healthcare policies and practices that focused resource on the pandemic response created a particularly precarious situation for people with life-shortening conditions who face accelerated premature death when their basic health needs are not met. Some participants highlighted how this made them feel insecure and invisible.

#### Disruption of Typical Care Arrangements

Young adults with life-shortening conditions live precariously because they often rely on others to meet their basic needs. Relying on others in order to stay alive is to be in a fundamentally precarious embodied state necessitating the management of numerous interdependencies. At a minimum this might include occasional support with housework, grocery shopping or some aspects of personal care. More often young adults will require 24-hour care that includes the complex management of medical technologies or treatments (Cook et al., 2016). During the pandemic the need to have people coming in and out of one’s home in order to receive care increased the risk of Coronavirus transmission for those who were shielding. In many cases, this was an unwanted but unavoidable risk. Participants did everything they could to minimise and manage the risks while also often expressing gratitude and concern for those who supported them. In a small number of cases participants were refused care but more typically young adults negotiated with their care teams and relied more on the family for care, particularly mothers. There was also a notable geographical pivot towards the family to meet both immediate and future care needs.

#### Strengths and Limitations

This study has strengths and limitations. It makes an important contribution to understanding the nature of precarity for those who are corporeally vulnerable and who already live precarious lives. To our knowledge, this is the only qualitative research study focussing on the experiences of young adults with life-shortening conditions during the pandemic. We were fortunate to work within a large multi-disciplinary inclusive team; this added richness to our interpretation of the data and, as Charmaz (2014) proposes, we were able to reflect on our own subjective positionalities. We were privileged to be able to interview 26 young adults during a demonstrably difficult time. Some limitations are also present. In terms of the demographic characteristics of our study group we recruited more women and more white people and none of our young adult participants reported any significant cognitive impairment which is not necessarily representative of the population (Feudtner et al., 2011). This means that there will be experiences not addressed by our analysis. Given our sensitivity to the potential vulnerability of research participants and the practicalities of working in tandem within a large research team, theoretical sampling was challenging, and this means we may not have fully saturated some of our key analytical categories, with inevitable consequences for theory-building.

Common to all grounded theory our analytical focus emerged during the research process and is grounded in our data. Such research is therefore context and situation specific which some might suggest limits the transferability – or generalisability – of our findings. However, other scholars would argue that cGT studies provide new insights that are credible, original, resonant, useful and transferable to other similar research problems or fields (Charmaz & Thornberg, 2021).

## Conclusion and Implications for Practice

The study has demonstrated that participants contend with both the corporeal vulnerability that is applicable to all human life (Butler, 2004a) as well as a very particular embodied precariousness peculiar to those with a life-shortening condition. In this article we have sought to show how public health measures introduced to control the pandemic and keep people safe have intensified the precarity of this group. Being more aware of how language and behaviours that other and de-value the lives and experiences of people with life-shortening conditions is an important consideration for practitioners. It is important to recognise that this othering is acutely felt and can have a significant impact on emotional wellbeing and sense of self. Organisations that support young adults with such conditions may wish to consider the need for enhanced emotional support services, as individuals come to terms with their experiences of othering and the pandemic. The resolve to live was very strongly communicated by study participants but the precariousness of living with a life-shortening condition meant that individuals could quickly become more vulnerable. Information and training may be useful in supporting young adults to develop systems that keep them safe – not only during this and other pandemics – but also in the context of any future unexpected and unwanted change.

This research is part of a larger study which included interviews with family members who live with or support young adults with life-shortening conditions. Participants increasingly relied on others during the pandemic including formal and informal carers, pivoting strongly towards family (especially mothers) for care. Our findings indicate that further awareness and more research on the experiences and needs of professional carers and their own families as well as participants’ families would be valuable.
